# Predictive Factors of Cardiac Function Recovery and Mortality in Patients with Reduced Ejection Fraction Undergoing Transcatheter Aortic Valve Implantation

**DOI:** 10.3390/medicina61020266

**Published:** 2025-02-04

**Authors:** Murat Can Güney, Hakan Süygün, Melike Polat, Hüseyin Ayhan, Telat Keleş, Zeynep Şeyma Turinay Ertop, Betül Banu Karasu, Engin Bozkurt

**Affiliations:** 1 Department of Cardiology, Faculty of Medicine, Medicana International Ankara Hospital, Atılım University, Söğütözü, 2176. Sk. No: 3, Çankaya 06510, Turkey; melike.polat@medicana.com.tr; 2 Department of Cardiology, Faculty of Medicine, Karamanoglu Mehmetbey University, Karaman Training and Research Hospital, Karaman 70100, Turkey; hakansuygun@kmu.edu.tr; 3 Department of Cardiology, Faculty of Medicine, University of Health Sciences Gulhane, Sincan Training and Research Hospital, Ankara 06010, Turkey; huseyin.ayhan@sbu.edu.tr; 4 Department of Cardiology, Faculty of Medicine, Ankara Yıldırım Beyazıt University, Ankara Bilkent City Hospital, Ankara 06800, Turkey; telat.keles@ybu.edu.tr; 5 Department of Cardiology, Medicana International Ankara Hospital, Ankara 06510, Turkey; zeynep.ertop@medicana.com.tr (Z.Ş.T.E.); engin.bozkurt@medicana.com.tr (E.B.); 6 Department of Cardiology, Sincan Training and Research Hospital, Ankara 06010, Turkey; benginoglu@yahoo.com

**Keywords:** transcatheter aortic valve implantation, heart failure, reduced ejection fraction, aortic stenosis

## Abstract

*Background and Objectives*: Patients with reduced left ventricular ejection fraction (LVEF) are reported to have unfavorable outcomes following transcatheter aortic valve implantation (TAVI). This study aims to evaluate outcomes and identify predictive factors for LVEF recovery following TAVI in patients with reduced LVEF. *Materials and Methods*: This retrospective study analyzed 114 patients with symptomatic severe aortic stenosis (AS) with LVEF < 40% who underwent TAVI between 2011 and 2023 at two centers. Echocardiographic parameters, including LVEF, ventricular dimensions, and relative wall thickness (RWT), were assessed at baseline and during follow-up. The outcomes and predictors of substantial LVEF improvement and mortality were analyzed using univariate and multivariate logistic regression methods. *Results*: Anemia (OR = 4.345, 95% CI: 1.208–15.626, *p* = 0.024), RWT (OR = 1.224, 95% CI: 1.064–1.407, *p* = 0.005), and early post-procedural changes in left ventricular end-systolic dimension (LVESD) (OR = 1.297, 95% CI: 1.037–1.622, *p* = 0.023) and left ventricular end-diastolic dimension (LVEDD) (OR = 1.346, 95% CI: 1.034–1.753, *p* = 0.027) at one-month follow-up were identified as significant factors associated with LVEF recovery at one year. Regarding factors related to mortality, higher baseline AVMG levels were associated with a lower probability of death after one year (OR = 0.926, 95% CI: 0.875–0.979, *p* = 0.007). Conversely, a more limited increase in LVEF from baseline to the final follow-up was linked to poor prognosis and higher mortality at one year (95% CI: 1.045–1.594, *p* = 0.018). *Conclusions*: This study demonstrated that TAVI in patients with AS and reduced LVEF can be performed with high procedural success, low mortality, and significant improvement in cardiac function during follow-up. Additionally, anemia, baseline RWT, and early post-procedural changes in LVESD and LVEDD were identified as factors associated with LVEF recovery. Baseline AVMG and changes in LVEF at the final follow-up were found to be significant predictors of total mortality.

## 1. Introduction

Aortic stenosis (AS) is among the most prevalent valvular heart diseases, especially in elderly individuals, and its incidence is anticipated to rise with increasing life expectancy in the general population [1]. However, despite its success, a subset of transcatheter aortic valve implantation (TAVI) patients, especially among those with heart failure and reduced left ventricular ejection fraction (LVEF), face significant peri-procedural challenges [2]. Conservative management of this high-risk patient group is associated with poor prognosis. In contrast, surgical aortic valve replacement (SAVR) can improve survival and reverse left ventricular (LV) dysfunction, but carries a high risk of operative mortality [3]. As a less invasive procedure, TAVI offers a viable alternative treatment strategy to SAVR for AS patients with reduced LVEF [4]. There are conflicting results in the literature regarding whether low-baseline LVEF is associated with poor prognosis after TAVI. Some studies suggest that low-baseline LVEF has no significant impact on outcomes, while others report a strong association with poor prognosis. However, it has been consistently shown that LVEF recovery during follow-up after TAVI is associated with improved prognosis [5,6]. Clinical decision-making for these patients is challenging due to the lack of well-established predictors for TAVI outcomes. Additionally, the literature presents conflicting findings regarding predictors of LVEF recovery after TAVI. Identifying these predictors is essential for optimizing patient selection and improving survival rates.

The aim of this study is to identify the predictors of mortality and cardiac function recovery in patients with reduced LVEF undergoing TAVI.

## 2. Methods

### 2.1. Patient Population

This retrospective study analyzed data from symptomatic severe AS patients (aortic valve area < 1 cm^2^) who underwent TAVI at two centers between October 2011 and January 2023. Out of 610 consecutive patients who underwent TAVI at the participating centers, 114 patients with reduced LVEF (<40%) and complete clinical and echocardiographic data at baseline and follow-up were included in the outcome analysis. Patients without reduced LVEF or with significantly missing data at any visit were excluded. From the initial cohort, 210 patients were identified with an LVEF below 40%. Both centers involved in this study are tertiary referral centers specializing in valvular heart diseases, with a substantial proportion of patients coming from outside their local regions. As a result, follow-up care at local hospitals limits access to complete data, leading to numerous exclusions. Approval for the study was obtained from the local ethics committee of Ataturk Education and Research Hospital (Approval No: 2011-68, dated 18 March 2011), and the study was conducted in accordance with the Declaration of Helsinki guidelines for research involving human participants.

### 2.2. Definitions and Data Collection

Patient data were obtained from electronic databases of the institutions and individual patient records. Mortality information was gathered from the national service for health database or via telephone follow-ups. Follow-up data were acquired through scheduled hospital visits at approximately 1, 6, and 12 months post-procedure, during which comprehensive clinical evaluations, transthoracic echocardiographic assessments, and routine laboratory tests were conducted. Echocardiographic parameters were measured according to European Association of Cardiovascular Imaging (EACVI) guidelines. Anemia was defined based on the World Health Organization (WHO) criteria as a hemoglobin level of <13 g/dL for men and <12 g/dL for women [7].

### 2.3. Echocardiography

Transthoracic echocardiography was conducted using a Vivid 7 device (General Electric, Horten, Norway) and interpreted by two nationally certified, experienced echocardiographers. LVEF was assessed using the biplane Simpson method based on apical and two-chamber views. Changes in LVEF, left ventricular end-diastolic dimension (LVEDD), and left ventricular end-systolic dimension (LVESD) were assessed by comparing baseline and follow-up echocardiographic measurements. These changes were expressed as percentage differences, calculated as follows: delta LVEDD (d-LVEDD), delta LVESD (d-LVESD), and delta LVEF (d-LVEF). The formulas used for these calculations were defined previously [8]. All patients underwent thorough evaluation, including dobutamine stress echocardiography and aortic valve calcium scoring when indicated, to separate true severe AS from pseudo-severe AS. However, as a result of incomplete dobutamine stress echocardiography data, patients were not classified into low-flow, low-gradient (LF-LG) AS.

### 2.4. TAVI Procedure

The indication for TAVI and procedural specifics were established through a multidisciplinary evaluation by a heart team comprising a cardiologist, cardiac surgeon, and anesthesiologist. For procedural planning, all patients underwent contrast-enhanced multidetector computed tomography to assess anatomy and access routes. The transfemoral approach was universally adopted, and procedures were performed in a catheterization laboratory using fluoroscopic guidance, under either general anesthesia or deep sedation, following established protocols in the literature [9]. Edwards SAPIEN XT and SAPIEN 3 valves were used (Edwards Lifesciences, Irvine, CA, USA).

### 2.5. Statistical Analysis

Data analysis was performed using IBM SPSS Statistics version 25 (IBM Corporation, Armonk, NY, USA). The Kolmogorov–Smirnov test was used to assess the normality of data distribution, and Levene’s test was applied to evaluate the homogeneity of variances. Categorical variables were expressed as frequencies (n) and percentages (%), while continuous variables were presented as mean ± standard deviation (SD), median (min–max), or median [25th–75th percentiles], as appropriate. Whether the differences among follow-up times in terms of echocardiographic parameters were statistically significant or not was evaluated by repeated measurements of ANOVA via Wilks’ Lambda test or the Friedman test where applicable. When the *p*-values from Wilks’ Lambda or the Friedman test indicated statistical significance, the Bonferroni-adjusted multiple comparison test or the Dunn–Bonferroni test was applied to identify which follow-up time points differed from others. Univariate logistic regression analyses were conducted to determine the associations between the main outcome variables (i.e., at least ≥20% increase in LVEF levels at the end of the 1st year and mortality) and demographic and clinical features and laboratory measurements. To determine the best predictors on the main outcome, variables were investigated by multiple logistic regression analysis. Variables with a *p*-value < 0.10 in the univariate analysis were selected as candidate risk factors for inclusion in the multivariate logistic regression model. Variables indicating multicollinearity were carefully reviewed and excluded where necessary to maintain the validity and interpretability of the regression model. Odds ratios (OR), 95% confidence intervals, and Wald statistics for each independent variable were also calculated. A *p*-value of less than 0.05 was considered statistically significant.

## 3. Results

This study included 114 patients, with a mean age of 75.6 ± 8.8 years, of whom 64 (56.1%) were male. The median STS score was 8.3, and the mean baseline LVEF was 30.6 ± 7.6%. Over the course of the one-year follow-up period, 28 patients died, resulting in a total one-year mortality rate of 24.6%. Baseline characteristics and echocardiographic data are presented in Table 1 and Table 2, respectively.

**Figure 1:**
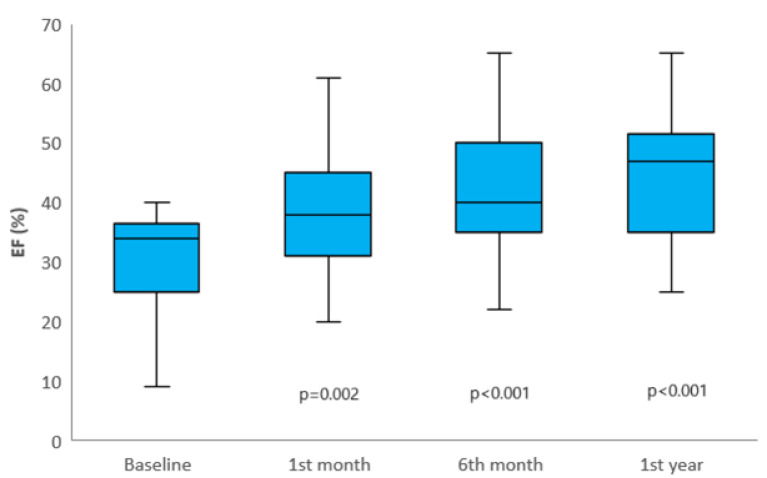
Changes in LVEF at 1 month, 6 months, and 1 year post-TAVI follow-up. Significant improvements were observed at each time point compared to baseline (*p* = 0.002 for 1 month, *p* < 0.001 for 6 months, and *p* < 0.001 for 1 year). The horizontal line within each box represents the median, while the upper and lower edges correspond to the 75th and 25th percentiles, respectively. The whiskers extending from the box indicate the maximum and minimum EF levels.

During follow-up, a significant difference was detected in median LVEF levels across visit times (*p* < 0.001). This difference was driven by progressively higher LVEF levels at 1 month, 6 months, and 1 year post-baseline (*p* = 0.002, *p* < 0.001, and *p* < 0.001, respectively). Additionally, LVEF levels at 6 months and 1 year were significantly higher compared to 1 month (*p* = 0.020 and *p* < 0.001, respectively), and LVEF levels at 1 year were significantly higher than those at 6 months (*p* < 0.001). These findings are illustrated in Figure 1. There was a statistically significant difference in LVEDD levels across follow-up times (*p* < 0.001), primarily due to lower LVEDD levels observed at 1 month and 6 months post-baseline (*p* < 0.001). Additionally, LVEDD levels at 6 months were significantly lower than those at 1 month (*p* < 0.001). Similarly, a significant difference in median LVESD levels was observed across follow-up times (*p* < 0.001), primarily due to lower LVESD levels at 1 and 6 months post-baseline (*p* < 0.001). Furthermore, LVESD levels at 6 months were significantly lower than those at 1 month (*p* < 0.001), as shown in Table 3.

This study aimed to identify factors associated with changes in LVEF one year after TAVI. The results of the univariate logistic regression analysis for potential predictors of LVEF change at one year are presented in Supplementary Table S1. All variables with *p* < 0.10 in the univariate analysis were included as candidate risk factors in the multivariate logistic regression model. The results of this analysis, which examined the combined effects of all potential predictors of changes in LVEF levels at one year of follow-up, are presented in Table 4. The most significant factors predicting the likelihood of less than a 20% increase in LVEF after one year of follow-up were the presence of anemia, baseline LVEF, baseline RWT, and changes in LVESD and LVEDD during the first month. Patients without a history of anemia had a significantly higher likelihood of achieving more than a 20% increase in LVEF after one year of follow-up compared to those with a history of anemia (OR = 4.345, 95% CI: 1.208–15.626, *p* = 0.024). Each 0.01-unit decrease in baseline RWT significantly increased the likelihood of achieving less than a 20% increase in LVEF after one year of follow-up (OR = 1.224, 95% CI: 1.064–1.407, *p* = 0.005). After adjusting for other factors, smaller percentage decreases in LVESD (OR = 1.297, 95% CI: 1.037–1.622, *p* = 0.023) and LVEDD (OR = 1.346, 95% CI: 1.034–1.753, *p* = 0.027) at the end of the first month were significantly associated with a higher likelihood of a less than 20% increase in LVEF after one year of follow-up.

Supplementary Table S2 presents the findings of the univariate logistic regression analysis for factors potentially predicting mortality after one year of follow-up. All variables with *p* < 0.10 in the univariate analysis were included as candidate risk factors in the multivariate regression model. Table 5 presents the results of the multivariate logistic regression analysis, which evaluates the combined effects of all factors that could potentially differentiate between the survivors and those who died within one year of follow-up. The most significant factors distinguishing between survivors and those who died within one year of follow-up were the baseline aortic valve mean gradient (AVMG) and the change in LVEF at the final follow-up. Higher baseline AVMG levels were significantly associated with a lower likelihood of death after one year of follow-up (OR = 0.926, 95% CI: 0.875–0.979, *p* = 0.007). Additionally, every 10% smaller increase in LVEF at the last follow-up compared to baseline significantly increased the likelihood of mortality after one year by 1.291 times (95% CI: 1.045–1.594, *p* = 0.018). For patients who survived, LVEF at one year was evaluated, while for those who died, the LVEF at their last follow-up visit was considered.

## 4. Discussion

This study demonstrates that TAVI can be successfully performed in a large cohort of patients with reduced LVEF, achieving relatively low mortality rates consistent with findings reported in the literature. The results highlight the significance of anemia, baseline RWT, and early post-procedural changes in LVESD and LVEDD at one-month follow-up as key factors associated with LVEF recovery after one year. Furthermore, the analysis identified baseline AVMG and changes in LVEF at the final follow-up as critical predictors of total cumulative mortality at one year.

More than half of TAVI candidates present with pre-procedural anemia, predominantly attributed to iron deficiency [10]. This condition has been associated with poor clinical outcomes and reduced functional capacity following TAVI [11]. Notably, in this group with low LVEF, lower hemoglobin levels were associated with higher rates of heart failure rehospitalization and increased global and cardiac mortality. Furthermore, studies have shown that heart failure patients with anemia and iron deficiency experience worse outcomes, although intravenous iron therapy has been demonstrated to improve their clinical status [7]. In the TOPAS-TAVI study, anemia was identified as a predictor of all-cause mortality in patients with low-flow, low-gradient aortic stenosis undergoing TAVI [12]. Additional data from this study on the relationship between anemia and reduced LVEF recovery highlight a critical aspect of patient management in the context of heart failure and TAVI. Anemia exacerbates myocardial ischemia, further impairing cardiac function and potentially affecting LVEF recovery. Addressing anemia preprocedurally through interventions such as iron supplementation or other targeted therapies could enhance cardiac recovery and improve patient outcomes. Incorporating routine anemia screening and treatment into pre-TAVI evaluations may represent an important step in optimizing patient management.

AS leads to an increase in LV pressure, hypertrophic remodeling of the LV, and an increase in relative wall thickness [13]. The findings from this study, which demonstrate that higher baseline RWT is associated with better LVEF recovery in AS patients with low LVEF undergoing TAVI, challenge the traditional view in the literature that concentric remodeling, characterized by higher RWT, is an unfavorable factor in AS patients [14]. These findings may suggest a more detailed understanding of the cardiac remodeling process in AS patients with reduced ventricular function. Traditionally, concentric remodeling, marked by an increase in RWT, has been viewed as a maladaptive response to pressure overload, often resulting in impaired cardiac function [15]. However, in the specific subset of patients with low EF, a higher basal RWT might indicate a residual adaptive capability of the heart to maintain contractile function. In contrast to traditional views that associate higher basal RWT with adverse outcomes in aortic stenosis, our results reveal that, in patients with reduced ventricular function, a higher basal RWT correlates with better recovery of LVEF post-TAVI [16]. This finding emphasizes the need for a better understanding of cardiovascular remodeling and its prognostic implications, encouraging further research to optimize outcomes, especially in the sub-group of a reduced ventricular function patient population undergoing TAVI procedures. These insights could guide patient selection and procedural planning by identifying individuals who are more likely to benefit from TAVI.

This study examined the relationship between changes in LVEDD and LVESD during the first month post-TAVI and their ability to predict LVEF recovery within the first year. Previous studies have demonstrated the impact of early LVEF recovery immediately after the procedure on patient survival, underscoring the importance of left ventricular reverse remodeling as a favorable prognostic factor [5]. Our study extends this understanding by examining left ventricular dimension changes at the one-month point. It was observed that decreases in LVEDD and LVESD during this period could serve as significant indicators of LVEF recovery over the first year following TAVI. These findings may help clinicians identify patients with a lower likelihood of LVEF recovery early. Furthermore, the relationship between early changes in LVEDD and LVESD and one-year LVEF recovery offers insights into the underlying mechanism of EF recovery after TAVI, emphasizing the role of reverse remodeling rather than simply the reduction in pressure overload. This understanding can inform future research aimed at further exploring the remodeling process and its implications for long-term outcomes in TAVI patients. Additionally, consistent with our findings, the literature also highlights an inverse relationship between baseline LVEF and the magnitude of LVEF improvement one year after TAVI [17,18]. Lower baseline LVEF is associated with a more pronounced recovery of ventricular function, highlighting the potential for significant cardiac function restoration in patients with severe systolic impairment prior to TAVI. This study contributes to the existing literature on the safety and efficacy of TAVI in this high-risk patient cohort with reduced LVEF, further supporting the viability of TAVI as a therapeutic intervention to improve cardiac outcomes and survival in this challenging clinical context.

Predictors of LVEF recovery after TAVI have been extensively explored in the literature. Factors such as lower baseline LVEF, absence of prior coronary artery bypass grafting (CABG), preserved stroke volume index (SVI), absence of atrial fibrillation (AF), no history of myocardial infarction (MI), estimated glomerular filtration rate (GFR) >60 mL/min/1.73 m^2^, and higher baseline mean aortic valve gradient have all been associated with favorable LVEF improvement [6,12,19]. This study highlights the combined significance of anemia and baseline RWT as factors associated with LVEF recovery, variables that have been less extensively explored in prior research. Additionally, this study diverges from prior research by evaluating one-month changes in LVEDD and LVESD as predictors of long-term LVEF recovery, providing insights into the short-term remodeling process and its role in cardiac recovery. This study emphasizes the importance of integrating systemic factors like anemia and short-term ventricular remodeling parameters to optimize outcomes in TAVI patients. These findings may encourage further research into targeted preprocedural and early postprocedural interventions.

In this study, although patients were not categorized as low-flow, low-gradient or normal-flow AS, a higher baseline AVMG was found to be associated with a lower mortality rate in patients undergoing TAVI. This finding suggests that patients with more significant hemodynamic obstruction at baseline may have a more favorable prognosis post-TAVI, aligning with reports in the existing literature [20]. Additionally, a direct correlation was observed between the magnitude of LVEF improvement at the last follow-up after TAVI and mortality rates, with greater improvements in LVEF being associated with lower mortality. A 10% increase in LVEF was selected as the threshold for mortality analyses based on prior research demonstrating its predictive value for mortality after TAVI, with a sensitivity of 50%, specificity of 75%, and an AUC of 0.72. This cutoff reflects significant left ventricular recovery and its association with improved survival. While that study previously demonstrated the prognostic significance of early LVEF improvement after TAVI, this study appears to be among the first to establish a direct relationship between LVEF recovery at late follow-up and total one-year cumulative mortality [5]. These findings highlight the critical importance of both the initial hemodynamic burden, as indicated by the aortic gradient, and the capacity for cardiac function recovery, as reflected by changes in LVEF, in determining patient outcomes after TAVI.

## 5. Limitations

Our study has several limitations that warrant acknowledgment. The first limitation is the retrospective nature of the study, which inherently restricts the ability to control for certain variables and relies on previously collected data. Secondly, SVI and dobutamine stress echocardiographic data, critical determinants in assessing the severity of AS and guiding therapeutic decisions, were not calculated. This omission precluded our ability to classify patients into low-flow or normal-flow AS groups, an essential distinction that influences prognosis and treatment strategy. Additionally, the lack of follow-up data on RWT limited our capacity to analyze changes in left ventricular geometry over time, a factor that can provide valuable insights into disease progression or regression post-intervention. These limitations underscore the need for a comprehensive evaluation of hemodynamic parameters and cardiac remodeling in future research to enhance our understanding of aortic stenosis and its management.

## 6. Conclusions

In this study, we found that anemia and basal RWT, early post-procedural changes in LVESD and LVEDD, have been identified as significant factors related to one-year LVEF recovery. Additionally, our findings show the predictive value of the baseline AVMG and changes in LVEF at the last follow-up as determinants of total cumulative one-year mortality in this patient population.

## Figures and Tables

**Table 1:** Basal characteristics of the study population.

Parameters	*n* = 114
Age	75.6 ± 8.8
Gender (Male)	64 (56.1%)
BMI (kg/m^2^)	26.9 ± 4.7
STS score	8.3 (2.6–28.4)
CAD	46 (40.4%)
CABG	36 (31.6%)
NYHA	
II	6 (5.3%)
III	74 (64.9%)
IV	34 (29.8%)
CVA	6 (5.3%)
PAD	46 (40.4%)
COPD	69 (50.5%)
Type 2 DM	44 (38.6%)
HT	98 (86.0%)
HL	51 (44.7%)
Baseline GFR	66.7 ± 16.1
AF	41 (36.0%)
Anemia	57 (50.0%)
Valve Type	
Edwards Sapien XT	95 (83.3%)
SAPIEN 3	19 (16.7%)
One-year cumulative total mortality	28 (24.6%)

BMI: body mass index; STS: the Society of Thoracic Surgery; CAD: coronary artery disease; CABG: coronary artery bypass graft; NYHA: New York Heart Association; CVA: cerebrovascular accident; PAD; peripheric artery disease; COPD: chronic obstructive pulmonary artery disease; DM: diabetes mellitus; HT: hypertension; HL: hyperlipidemia GFR: glomerular filtration rate; AF: atrial fibrillation.

**Table 2:** Basal echocardiographic data of the study population.

Parameters	*n* = 114
MR (Moderate–Severe)	21 (18.4%)
LVEF (%)	30.6 ± 7.6
LVEDD (cm)	5.5 ± 0.64
LVESD (cm)	4.3 ± 0.80
Septal Wall Thickness (cm)	1.24 ± 0.23
Posterior Wall Thickness (cm)	1.17 ± 0.20
RWT	0.44 ± 0.10
LVMI (g/m^2^)	158.5 ± 36.6
AVA (cm^2^)	0.67 ± 0.19
sPAP (mmHg)	49.4 ± 15.8
AVMG (mmHg)	43.0 ± 3.4

MR: mitral regurgitation; LVEF: left ventricular ejection fraction; LVEDD: left ventricular end-diastolic dimension; LVESD: left ventricular end-systolic dimension; RWT: relative wall thickness; LVMI: left ventricular mass index; AVA: aortic valve area; sPAP: pulmonary artery pressure; AVMG: aortic valve mean gradient.

**Table 3:** Changes in the echocardiographic parameters through follow-up visits.

Parameters	Basal	1st Month	6th Month	1st Year	*p*
LVEF (%)	34.0 [25.0–36.5]	38.0 [31.0–45.0]	40.0 [35.0–50.0]	47.0 [35.0–51.5]	<0.001
LVEDD (cm)	5.44 ± 0.68	5.20 ± 0.68	5.09 ± 0.65	n/a	<0.001
LVESD (cm)	4.20 [3.70–4.82]	3.70 [3.17–4.52]	3.70 [3.10–4.20]	n/a	<0.001

LVEF: left ventricular ejection fraction; LVEDD: left ventricular end-diastolic dimension; LVESD: left ventricular end-systolic dimension.

**Table 4:** Multivariate logistic regression analysis of parameters for prediction of EF change in one year.

Parameters	OR	95% CI	Wald	*p*
STS score	0.879	0.690–1.119	1.096	0.295
COPD	2.337	0.652–8.377	1.698	0.193
Anemia	4.345	1.208–15.626	5.061	0.024
Basal LVEF	1.294	1.067–1.568	6.893	0.009
LVEDD change at first month	1.346	1.034–1.753	4.864	0.027
LVESD change at first month	1.297	1.037–1.622	5.200	0.023
Basal RWT	1.224	1.064–1.407	8.031	0.005

STS: The Society of Thoracic Surgery; COPD: chronic obstructive pulmonary artery disease; LVEF: left ventricular ejection fraction; LVEDD: left ventricular end-diastolic dimension; LVESD: left ventricular end-systolic dimension; RWT: relative wall thickness.

**Table 5:** Multivariate logistic regression analysis of parameters for the prediction of total mortality in one year.

Parameters	OR	95% CI	Wald	*p*
Age (years)	1.074	0.993–1.162	3.172	0.075
STS score	0.977	0.854–1.118	0.111	0.739
Basal GFR	0.992	0.954–1.032	0.160	0.689
LVEF change at the last visit (%)	1.291	1.045–1.594	5.633	0.018
Basal AVMG	0.926	0.875–0.979	7.202	0.007

STS: The Society of Thoracic Surgery; GFR: glomerular filtration rate; LVEF: left ventricular ejection fraction; AVMG: aortic valve mean gradient.

## Data Availability

The data presented in this study are available upon request from the corresponding author, as most of the data originate from the government social security system.

## Supplementary Materials

The following supporting information can be downloaded at: https://www.mdpi.com/article/10.3390/medicina61020266/s1, Table S1: Univariate logistic regression analysis of parameters for prediction of EF increase in one-year; Table S2: Univariate logistic regression analysis of parameters for prediction of total mortality in one-year.
