# Supplementary material for: Predictive Factors of Cardiac Function Recovery and Mortality in Patients with Reduced Ejection Fraction Undergoing Transcatheter Aortic Valve Implantation
Source: Medicina (Kaunas). 2025 Feb 4;61(2):266. doi: 10.3390/medicina61020266 (PMC11857224; doi:10.3390/medicina61020266)
Supplement: Supplementary file 1 [file medicina-61-00266-s001.zip › medicina-3451086-supplementary.pdf]

Supplement Table S1. Univariate logistic regression analysis of parameters for prediction of EF increase in one-year

Parameters	EF increase ≥%20 (n=62)	EF increase <%20 (n=24)	p	OR (95% CI)
Age	74.0±7.9	74.1±7.9	0.972	1.001 (0.942-1.063)
Female	24 (39.3%)	13 (54.2%)	0.217	1.822 (0.702-4.726)
BMI (kg/m^2)	27.5±4.3	26.6±4.2	0.386	0.950 (0.846-1.067)
STS score	10.1±4.7	7.9±3.2	0.043	0.867 (0.754-0.995)
CAD	26 (42.6%)	10 (41.7%)	0.936	0.962 (0.369-2.504)
CABG	19 (31.1%)	7 (29.2%)	0.858	0.910 (0.324-2.559)
NYHA class	3 [3-4]	3 [3-4]	0.974	1.015 (0.416-2.476)
CVA	3 (4.9%)	1 (4.2%)	0.883	0.841 (0.083-8.503)
PAD	27 (44.3%)	9 (37.5%)	0.571	0.756 (0.287-1.990)
COPD	2 [1-2]	2 [1.5-3]	0.055	1.803 (0.987-3.296)
Type 2 DM	23 (37.7%)	11 (45.8%)	0.492	1.398 (0.538-3.634)
HT	53 (86.9%)	21 (87.5%)	0.939	1.057 (0.255-4.371)
HL	29 (47.5%)	9 (37.5%)	0.403	0.662 (0.252-1.742)
AF	22 (36.1%)	9 (37.5%)	0.902	1.064 (0.400-2.828)
Anemia	24 (39.3%)	17 (70.8%)	0.011	3.744 (1.351-10.374)
Basal GFR	67.7±16.1	70.0±15.4	0.540	1.010 (0.979-1.041)
SAPIEN 3 valve implantation	10 (16.4%)	5 (20.8%)	0.630	1.342 (0.406-4.437)
Basal LVEF	30.0 [23.0-35.0]	35.0 [30.0-40.0]	0.016	1.096 (1.017-1.182)

LVEF change at first month	28.6 [13.4-45.5]	0.0 [-4.9 – 2.9]	<0.001	0.833 (0.762-0.911)
Basal LVEDD	5.4±0.7	5.6±0.5	0.104	1.797 (0.887-3.639)
LVEDD change at first month	-5.7±7.7	0.6±3.3	<0.001	1.224 (1.082-1.385)
LVEDD change at sixth month	-7.8±8.1	-0.5±4.1	<0.001	1.205 (1.083-1.340)
Basal LVESD	4.2 [3.6-4.7]	4.2 [3.8-5.0]	0.340	1.330 (0.741-2.388)
LVESD change at first month	-6.0 [-15.2 – -2.3]	0.0 [-2.5 – 2.7]	<0.001	1.268 (1.101-1.460)
LVESD change at sixth month	-13.5 [-21.1 – -8.3]	0.0 [-2.7 – 3.2]	<0.001	1.244 (1.120-1.383)
Basal septum wall thickness	1.3±0.2	1.2±0.2	0.260	0.283 (0.031-2.549)
Basal posterior wall thickness	1.2±0.2	1.2±0.2	0.368	0.323 (0.028-3.770)
Basal RWT	0.5±0.1	0.4±0.1	0.086	0.016 (0.000-1.815)
Basal LVMI	155.8±33.0	161.2±41.1	0.525	1.004 (0.991-1.018)
Basal AVA	0.7±0.2	0.7±0.2	0.735	1.535 (0.128-18.370)
Basal PAB	45.0 [35.0-65.0]	45.0 [35.0-58.7]	0.484	0.990 (0.961-1.019)
MR(Moderate-Severe)	12 (19.7%)	5 (20.8%)	0.904	1.075 (0.033-3.462)

Basal AVMG	42.0 [40.0-52.0]	45.0 [41.0-47.7]	0.706	0.990 (0.942-1.041)

BMI:body mass index; STS:The Society of Thoracic Surgery; CAD:coronary artery disease; CABG: coronary artery bypass greft; NYHA: New York Heart Association; CVO: cerebrovascular accident; PAD; peripheric artery disease; COPD: chronic obstructive pulmonary artery disease; DM: diabetes mellitus; HT: hypertension; HL:hyperlipidemia GFR:glomerular filtration rate; AF: atrial fibrillation; MR: mitral regurgitation; LVEF:left ventricular ejection fraction; LVEDD:left ventricular end-diastlic dimension; LVESD:left ventricular end-systolic dimension; RWT:relative wall thickness; LVMI:left ventricular mass index; AVA:aortic valve area; PAB:pulmonary artery pressure; AVMG: aortic valve mean gradient

Supplement Table S2. Univariate logistic regression analysis of parameters for prediction of total mortality in one-year

Parameters	Survive (n=86)	Exitus (n=28)	p	OR (95% CI)
Age	74.1±7.9	80.0±10.3	0.004	1.087 (1.027-1.151)
Female	37 (43.0%)	13 (46.4%)	0.753	1.148 (0.487-2.703)
BMI (kg/m^2)	27.2±4.2	25.8±5.8	0.161	0.932 (0.846-1.028)
STS score	9.5±4.4	12.4±7.5	0.021	1.093 (1.014-1.179)
CAD	36 (41.9%)	10 (35.7%)	0.565	0.772 (0.319-1.867)
CABG	26 (30.2%)	10 (35.7%)	0.588	0.780 (0.317-1.918)
NYHA class	3 [3-4]	3 [3-4]	0.960	1.020 (0.463-2.250)
CVA	4 (4.7%)	2 (7.1%)	0.611	1.577 (0.273-9.109)

PAD	37 (43.0%)	9 (32.1%)	0.310	0.627 (0.255-1.544)
COPD	2 [1-2]	2 [1-3]	0.165	1.440 (0.860-2.411)
Type 2 DM	35 (40.7%)	9 (32.1%)	0.421	0.690 (0.280-1.702)
HT	75 (87.2%)	23 (82.1%)	0.505	0.675 (0.212-2.143)
HL	39 (45.3%)	12 (42.9%)	0.818	0.904 (0.382-2.137)
AF	31 (36.0%)	10 (35.7%)	0.975	0.986 (0.405-2.399)
Anemia	41 (47.7%)	16 (57.1%)	0.385	1.463 (0.619-3.458)
Basal GFR	68.5±15.8	61.0±16.0	0.036	0.971 (0.946-0.998)
SAPIEN 3 valve implantation	15 (17.4%)	4 (14.3%)	0.698	0.789 (0.239-2.609)
Basal LVEF	34.5 [25.0-37.2]	30.0 [25.0-35.0]	0.543	0.983 (0.930-1.039)
LVEF change at the last visit (%)	44.0 [16.1-72.1]	20.0 [0.0-28.6]	0.002	0.973 (0.956-0.990)
Basal LVEDD	5.4±0.7	5.6±0.5	0.170	1.597 (0.818-3.115)
LVEDD change at first month	-3.9±7.3	-2.2±6.6	0.300	1.037 (0.968-1.111)
LVEDD change at sixth month	-5.7±7.8	-11.8±7.3	0.137	0.922 (0.828-1.026)
Basal LVESD	4.2 [3.7-4.8]	4.5 [3.7-4.9]	0.356	1.286 (0.754-2.194)
LVESD change at first month	-3.2 [-8.7 – 0.0]	-2.0 [-8.8 – 2.3]	0.510	1.014 (0.974-1.055)
LVESD change at sixth month	-10.3 [-18.0 – -2.6]	-22.6 [-24.4 – -12.8]	0.164	0.949 (0.882-1.022)

Basal septum wall thickness	1.2±0.2	1.2±0.3	0.947	1.064 (0.172-6.578)
Basal posterior wall thickness	1.2±0.2	1.1±0.2	0.325	0.314 (0.031-3.160)
Basal RWT	0.4±0.1	0.4±0.1	0.118	0.028 (0.000-2.466)
Basal LVMI	157.0±35.2	163.3±40.8	0.430	1.005 (0.993-1.016)
Basal AVA	0.7±0.2	0.7±0.2	0.983	0.976 (0.105-9.061)
Basal PAB	45.0 [35.0-60.0]	50.0 [40.5-60.0]	0.549	1.008 (0.982-1.036)
MR (Moderate-Severe)	18 (20.9%)	3 (10.7%)	0.235	0.453 (0.123-1.672)
Basal AVMG	42.5 [40.0-51.2]	35.5 [24.7-48.0]	0.002	0.933 (0.892-0.976)

BMI:body mass index; STS:The Society of Thoracic Surgery; CAD:coronary artery disease; CABG: coronary artery bypass greft; NYHA: New York Heart Association; CVO: cerebrovascular accident; PAD; peripheric artery disease; COPD: chronic obstructive pulmonary artery disease; DM: diabetes mellitus; HT: hypertension; HL:hyperlipidemia GFR:glomerular filtration rate; AF: atrial fibrillation; MR: mitral regurgitation; LVEF:left ventricular ejection fraction; LVEDD:left ventricular end-diastlic dimension; LVESD:left ventricular end-systolic dimension; RWT:relative wall thickness; LVMI:left ventricular mass index; AVA:aortic valve area; PAB:pulmonary artery pressure; AVMG: aortic valve mean gradient
